# Thrombotic thrombocytopenia associated with COVID-19 infection or vaccination: Possible paths to platelet factor 4 autoimmunity

**DOI:** 10.1371/journal.pmed.1003648

**Published:** 2021-05-24

**Authors:** Michel Goldman, Cédric Hermans

**Affiliations:** 1 Institute for Interdisciplinary Innovation in Healthcare, Université libre de Bruxelles (ULB), Brussels, Belgium; 2 Division of Hematology, Hemostasis and Thrombosis Unit, Saint-Luc University Hospital, Université catholique de Louvain (UCLouvain), Brussels, Belgium

## Abstract

Michel Goldman and Cédric Hermans discuss thrombotic mechanisms in COVID-19 and rare adverse reactions to SARS-CoV-2 vaccinations.

## Introduction

Thrombotic thrombocytopenia mimicking heparin-induced thrombocytopenia has been observed in patients with severe Coronavirus Disease 2019 (COVID-19) or after immunisation with adenoviral vector-based vaccines against Severe Acute Respiratory Syndrome Coronavirus 2 (SARS-CoV-2). Herein, we discuss the pathogenesis of the autoimmune response to platelet factor 4 (PF4) that underlies these disorders.

There is convincing evidence that autoimmunity is involved in the pathogenesis of COVID-19 [1,2]. Regarding the severe forms of the disease in which thromboinflammation is prominent, both endothelial cells and platelets might be affected by autoimmune reactions in addition to direct viral infection and cytokine-mediated activation [3,4]. Firstly, multiple anti-phospholipid antibodies have been detected in the blood of hospitalized patients in relation with the severity of the disease and the formation of neutrophil extracellular traps known to contribute to thrombotic events [5]. A recent study further established that among anti-phospholipid autoantibodies detected in COVID-19 patients, immunoglobulin G (IgG) to cardiolipin and phosphatidylserine/prothrombin might be the ones driving endothelial cell activation [6]. In addition, anti-annexin A2 autoantibodies found in critically ill patients were suggested to contribute to small vessel damage in the lungs [7].

Besides endothelial cell damage, activation of platelets is the other cornerstone of the prothrombotic state characteristic of COVID-19 [4]. Several factors are involved including mitochondrial disturbances caused by hypoxia, mediators of inflammation, and other stressors, leading to platelet hyperactivation and apoptosis [4,8]. Furthermore, infection of platelets by the SARS-CoV-2 virus might also contribute to their activation via angiotensin converting enzyme 2 (ACE2)-dependent [9] as well as non-ACE2 mechanisms involving heparan sulfate [10] or CD147 [11]. Following viral entry, SARS-CoV-2 ssRNA might trigger intracellular Toll-like receptor 7–dependent activation pathways as in the case of influenza infection [12]. Antibody-mediated mechanisms involving engagement of the FcγRIIA receptor on platelets were also shown to contribute to procoagulant activity in severe COVID-19 [13,14]. Although the antigenic specificity of these antibodies could not always be defined, antibodies to PF4 were shown to be involved in certain cases [15–22].

PF4, also called CXCL4, is a tetrameric chemokine stored in platelet alpha-granules [23]. Upon platelet activation, PF4 is released and binds polyanions with high affinity [24]. Indeed, PF4 was shown to play a critical role in heparin-induced thrombocytopenia [25]. Below, we summarize the key features of heparin-induced thrombocytopenia before proposing that COVID-19 and adenovirus-vectored COVID-19 vaccines can on rare occasions cause autoimmune thrombotic thrombocytopenia mimicking heparin-induced thrombocytopenia.

### PF4 autoimmunity in heparin-induced thrombocytopenia

Heparin-induced thrombocytopenia is a severe prothrombotic condition that occurs in less than 5% of patients receiving heparin. Anti-PF4 antibodies are key biomarkers of heparin-induced thrombocytopenia [25]. They recognize an epitope exposed on PF4 tetramers upon conformational changes induced by their interaction with heparin or other long polyanions [26]. Indeed, injection of heparin has been shown to induce the release of PF4 [27], resulting in the assembly of PF4/heparin complexes, which activate complement and bind circulating B lymphocytes in a complement-dependent manner [28]. B cells responsible for the synthesis of PF4 autoantibodies display unique characteristics that enable them to rapidly mount an IgG response following a first exposure to heparin [29]. Indeed, B cells, which are able to produce anti-PF4 antibodies, are present in healthy individuals but in an anergic state that normally prevents their activation. This B cell tolerance might be broken upon heparin exposure and under some inflammatory conditions [30]. In these situations, anti-PF4 IgG antibodies elicit thrombus formation and thrombocytopenia via multiple mechanisms. Immune complexes assembled with PF4 bound to heparin induce platelet activation and aggregation by cross-linking FcγRIIA receptors [25]. Anti-PF4 antibodies also activate the procoagulant activity of monocytes by cross-linking their FcγRI receptors and of endothelial cells via the recognition of PF4 firmly attached to surface proteoglycans (PGs) [31]. Thrombocytopenia results from enhanced apoptosis and clearance of antibody-coated platelets in addition to consumption in the coagulation process [8].

A prothrombotic syndrome with all the features of heparin-induced thrombocytopenia has been reported in the absence of heparin exposure [32]. These observations led to the definition of a so-called “spontaneous heparin-induced thrombocytopenia” caused by anti-PF4 autoantibodies elicited by polyanions reproducing the conformational changes induced in PF4 tetramers by heparin [33]. Potential polyanions triggering “spontaneous heparin-induced thrombocytopenia” include bacterial wall components, nucleic acid materials, or endogenous PGs released by damaged cells.

### Thrombotic thrombocytopenia during COVID-19: An autoimmune reaction induced by SARS-CoV-2?

The high incidence of thrombotic and thromboembolic events during severe COVID-19 results in the frequent administration of heparin in affected patients [34]. Thrombosis can develop in unusual locations such as cerebral venous sinuses [35]. When thrombocytopenia develops in this setting, heparin-induced thrombocytopenia must be considered as a possible cause [18]. Indeed, several studies report the presence of anti-PF4/heparin antibodies in COVID-19 patients. However, these antibodies sometimes occur in absence of heparin administration [18]. Furthermore, they do not always activate platelets in presence of heparin/PF4 complexes [36], although they do so in presence of PF4 alone [14], suggesting that they were induced by another mechanism than classical heparin-induced thrombocytopenia [26]. There is indeed clinico-biological evidence that infection with SARS-CoV-2 by itself can elicit antibody-mediated thrombotic thrombocytopenia. IgG antibodies present in the serum of severe COVID-19 patients were found to induce platelet apoptosis and procoagulant activity via FcγRIIA receptor-dependent mechanisms [13]. The antigenic specificity of these antibodies was not defined, but one can speculate that at least some of them are directed against PF4.

**Fig 1:**
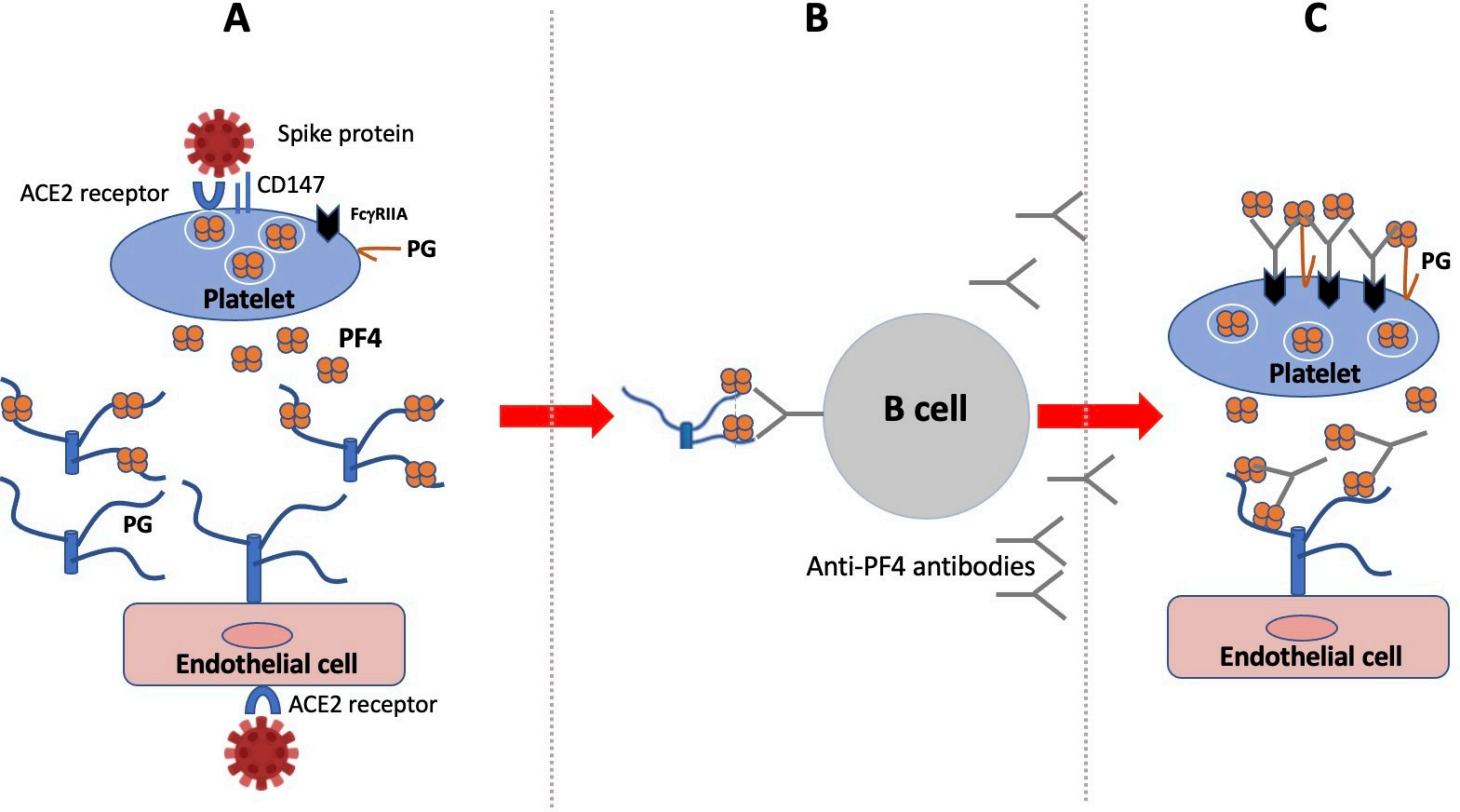
Hypothetical model for thrombotic thrombocytopenia during COVID-19. (A) SARS-CoV-2 induces the release of PF4 by activated platelets and of polyanionic PG by endothelial cells (e.g., syndecan and endocan). (B) Complexes of PF4 and PG expose PF4 immunogenic epitopes, which activate extrafollicular B lymphocytes secreting PF4 autoantibodies. (C) PF4 autoantibodies bind complexes of PF4 and PG on platelets and endothelial cells and stimulate their procoagulant activities. Cross-linking of FcγRIIA receptors also promote apoptosis and clearance of antibody-decorated platelets. COVID-19, Coronavirus Disease 2019; PF4, platelet factor 4; PG, proteoglycan; SARS-CoV-2, Severe Acute Respiratory Syndrome Coronavirus 2.

The model that we are proposing in Fig 1 is first based on the hyperactivation of platelets during COVID-19, resulting in the release of PF4 in the circulation [37]. Circulating PF4 could form complexes with endogenous polyanionic PGs released by damaged endothelial cells. Syndecan-1 and endocan are potential PG candidates since their serum levels are increased in severely ill COVID-19 patients in association with other markers of endothelial injury [38–40]. Complexes formed between PF4 and endothelial cell-derived polyanionic PG would then stimulate extrafollicular B cells producing anti-PF4 antibodies. Indeed, autoimmune responses elicited by extrafollicular B cells were previously suggested to be involved in the pathophysiology of severe COVID-19 [41]. Anti-PF4 antibodies would then recapitulate the sequence of events responsible for heparin-induced thrombocytopenia. Besides anti-PF4 autoantibodies, anti-phospholipid antibodies could also contribute to platelet activation as well as antiviral antibodies as observed in other infections [42,43].

### Thrombotic thrombocytopenia following COVID-19 vaccination

Several observations of prothrombotic thrombocytopenic events following vaccination with the adenovirus-vectored vaccine ChAdOx1 nCoV-19 vaccine (Vaxzevria, Oxford/AstraZeneca) were reported in European countries [44–46]. The incidence of these events is very low (around 1 in 100,000 recipients) but still significant by comparison with the background rate. As the clinical presentation is often reminiscent of heparin-induced thrombocytopenia, the hypothesis of a vaccine-induced autoimmune response to PF4 was put forward. Indeed, Greinacher and colleagues, Schultz and colleagues, and Scully and colleagues reported the detection of platelet-activating anti-PF4 antibodies in sera of patients suffering from unusual thrombotic events associated with thrombocytopenia within 4 to 16 days after injection of the ChAdOx1 nCoV-19 vaccine [44–46]. Shortly after these observations, 17 cases of thrombocytopenic thromboses affecting cerebral venous sinuses were reported in the United States after administration of the Ad26.COV2.S vaccine (Janssen/Johnson & Johnson), another adenoviral vector-based COVID-19 vaccine [47]. Strikingly, serum anti-PF4 antibodies were present in the 11 patients in whom they were searched for [47]. So far, there is no evidence for an increased incidence of similar events after administration of mRNA vaccines, suggesting a role for the adenoviral vectors in the induction of the anti-PF4 autoimmune response.

**Fig 2:**
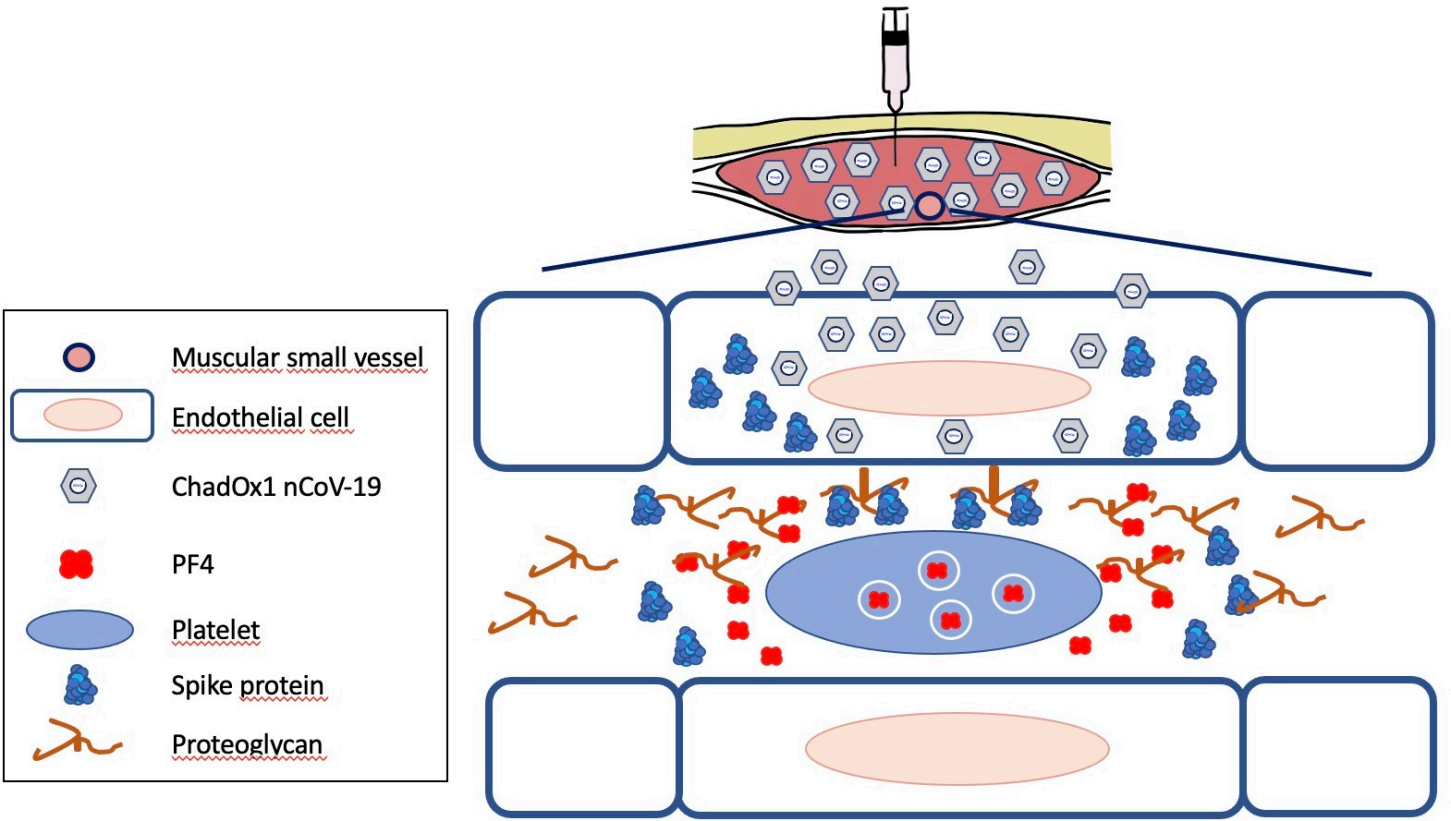
Hypothetical model for thrombotic thrombocytopenia after adenoviral vector-based COVID-19 vaccines. After intramuscular injection, vaccine adenoviruses infect endothelial cells, inducing their production of the SARS-CoV-2 Spike protein. Heparan sulfate PG could bind the spike protein on the luminal side of endothelial cells or be released by damaged cells. Spike proteins would activate platelets via ACE2-dependent and ACE2-independent mechanisms. PF4 released by activated platelets would become immunogenic after binding heparan sulfate PG shed from endothelial cells. ACE2, angiotensin converting enzyme 2; COVID-19, Coronavirus Disease 2019; PF4, platelet factor 4; PG, proteoglycan; SARS-CoV-2, Severe Acute Respiratory Syndrome Coronavirus 2.

Indeed, Greinacher and colleagues recently reported that ChAdOx1 nCoV-19 vaccine-induced anti-PF4 antibodies do not cross-react with the SARS-CoV-2 spike protein, excluding a phenomenon of molecular mimicry between the viral protein and PF4 [48]. The same group formulated several hypotheses about the vaccine components that could be involved, including adenovirus-derived substances [49]. As adenoviruses are known to activate platelets [50], it is plausible that the replication-deficient adenoviral vector could be directly responsible for the release of platelet-derived PF4. However, this hypothesis implies that significant amounts of vaccine particles would reach the bloodstream after intramuscular injection, which seems unlikely. An alternative scenario depicted in Fig 2 would involve endothelial cells. Indeed, endothelial cells are efficiently transduced upon intramuscular injection [51]. Transduced endothelial cells might be directly damaged by the spike protein that they synthesize, as suggested by in vitro and in vivo observations [52,53]. Furthermore, endothelial cells might expose the spike protein on their luminal side, possibly bound to PG of the glycocalyx as heparan sulfate PGs were shown to be attachment factors for the spike protein [54]. Platelets might then be recruited and activated by the spike protein bound to endothelial cells [9]. PF4 released by activated platelets could combine with anionic PGs shed from endothelial cells. In such a scenario, both the adenovirus and the spike protein would contribute to the formation of immunogenic PF4 following vaccination with adenoviral vector-based COVID-19 vaccines.

## Concluding remarks

Autoantibodies to PF4 contribute to thrombotic thrombocytopenia, which occasionally occurs during COVID-19 or after vaccination with adenoviral vector-based vaccines against SARS-CoV-2. We propose that heparan sulfate PG shed from damaged endothelial cells contribute to making PF4 immunogenic. As far as postvaccine thrombotic events are concerned, it will be important to specify the role of the adenoviral vector in view of the current developments of other vaccines based on the same technology. Finally, further research is needed to identify the risk factors, which predispose rare individuals to these severe complications.

## Abbreviations

ACE2: angiotensin converting enzyme 2
COVID-19: Coronavirus Disease 2019
IgG: immunoglobulin G
PF4: platelet factor 4
PG: proteoglycan
SARS-CoV-2: Severe Acute Respiratory Syndrome Coronavirus 2

## References

[1] Winchester N, Calabrese C, Calabrese LH. The intersection of COVID-19 and autoimmunity: What is our current understanding? Pathog Immun. 2021;6:31–54. 10.20411/pai.v6i1.417 . 33969248 PMC8097827

[2] Ramos-Casals M, Brito-Zerón P, Mariette X. Systemic and organ-specific immune-related manifestations of COVID-19. Nat Rev Rheumatol. 2021; 1–18. 10.1038/s41584-020-00559-x . 33903743 PMC8072739

[3] Bonaventura A, Vecchié A, Dagna L, Martinod K, Dixon DL, Van Tassell BW, et al. Endothelial dysfunction and immunothrombosis as key pathogenic mechanisms in COVID-19. Nat Rev Immunol. 2021. 10.1038/s41577-021-00536-9 . 33824483 PMC8023349

[4] Gu SX, Tyagi T, Jain K, Gu VW, Lee SH, Hwa JM, et al. Thrombocytopathy and endotheliopathy: crucial contributors to COVID-19 thromboinflammation. Nat Rev Cardiol. 2020;18(3):194–209. 10.1038/s41569-020-00469-1 . 33214651 PMC7675396

[5] Zuo Y, Estes SK, Ali RA, Gandhi AA, Yalavarthi S, Shi H, et al. Prothrombotic autoantibodies in serum from patients hospitalized with COVID-19. Sci Transl Med. 2020;12(570):eabd3876. 10.1126/scitranslmed.abd3876 . 33139519 PMC7724273

[6] Shi H, Zuo Y, Gandhi AA, Sule G, Yalavarthi S, Gockman K, et al. Endothelial cell-activating antibodies in COVID-19. MedRxiv. 2021. [preprint]. https://www.medrxiv.org/content/10.1101/2021.01.18.21250041v2 35174669 10.1002/art.42094 PMC9082472

[7] Zuniga M, Gomes C, Carsons SE, Bender MT, Cotzia P, Miao QR, et al. Autoimmunity to the lung protective phospholipid-binding protein annexin A2 predicts mortality among hospitalized COVID-19 patients. medRxiv. 2021. [preprint]. 10.1101/2020.12.28.20248807 PMC8859972 34244321

[8] Koupenova M, Freedman JE. Platelets and COVID-19: Inflammation, hyperactivation and additional questions. Circ Res. 2020;127(11):1419–21. 10.1161/CIRCRESAHA.120.318218 . 33151798 PMC7641185

[9] Zhang S, Liu Y, Wang X, Yang L, Li H, Wang Y, et al. SARS-CoV-2 binds platelet ACE2 to enhance thrombosis in COVID-19. J Hematol Oncol. 2020;13:1–22. 10.1186/s13045-019-0838-y . 32887634 PMC7471641

[10] Clausen TM, Sandoval DR, Spliid CB, Pihl J, Perrett HR, Painter CD, et al. SARS-CoV-2 infection depends on cellular heparan sulfate and ACE2. Cell. 2020;183:1043–57. 10.1016/j.cell.2020.09.033 . 32970989 PMC7489987

[11] Wang K, Chen W, Zhou Y-S, Lian J-Q, Zhang Z, Du P, et al. SARS-CoV-2 invades host cells via a novel route: CD147-spike protein. bioRxiv. 2020. [preprint]. 10.1101/2020.03.14.988345

[12] Koupenova M, Corkrey HA, Vitseva O, Manni G, Pang CJ, Clancy L, et al. The role of platelets in mediating a response to human influenza infection. Nat Commun. 2019; 10.1038/s41467-019-09607-x . 30992428 PMC6467905

[13] Althaus K, Marini I, Zlamal J, Pelzl L, Singh A, Häberle H, et al. Antibody-induced procoagulant platelets in severe COVID-19 infection. Blood. 2020;137(8):1061–71. 10.1182/blood.2020008762 . 33512415 PMC7791311

[14] Nazy I, Jevtic SD, Moore JC, Huynh A, Smith JW, Kelton JG, et al. Platelet-activating immune complexes identified in critically ill COVID-19 patients suspected of heparin-induced thrombocytopenia. J Thromb Haemost. 2021;19(5):1342–1347. 10.1111/jth.15283 . 33639037 PMC8014456

[15] Parzy G, Daviet F, Puech B, Sylvestre A, Guervilly C, Porto A, et al. Venous thromboembolism events following venovenous extracorporeal membrane oxygenation for severe acute respiratory syndrome coronavirus 2 based on CT scans. Crit Care Med. 2020;48(10):E971–E975. 10.1097/CCM.0000000000004504 . 32618700 PMC7328443

[16] May JE, Siniard RC, Marques M. The challenges of diagnosing heparin-induced thrombocytopenia in patients with COVID-19. Res Pract Thromb Haemost. 2020;4(6):1066–1067. 10.1002/rth2.12416 32838112 PMC7404754

[17] Riker RR, May TL, Fraser GL, Gagnon DJ, Bandara M, Zemrak WR, et al. Heparin-induced thrombocytopenia with thrombosis in COVID-19 adult respiratory distress syndrome. Res Pract Thromb Haemost. 2020;4(5):936–41. 10.1002/rth2.12390 . 32685905 PMC7276726

[18] Liu X, Zhang X, Xiao Y, Gao T, Wang G, Wang Z, et al. Heparin-induced thrombocytopenia is associated with a high risk of mortality in critical COVID-19 patients receiving heparin-involved treatment. medRxiv. 2020. [preprint]. 10.1101/2020.04.23.20076851

[19] Lingamaneni P, Gonakoti S, Moturi K, Vohra I, Zia M. Heparin-induced Thrombocytopenia in COVID-19. J Investig Med High Impact Case Rep. 2020; 10.1177/2324709620944091 . 32720827 PMC7388103

[20] Patell R, Khan AM, Bogue T, Merrill M, Koshy A, Bindal P, et al. Heparin induced thrombocytopenia antibodies in Covid-19. Am J Hematol. 2020; 10.1002/ajh.25935 . 32658337 PMC7405086

[21] Daviet F, Guervilly C, Baldesi O, Bernard-Guervilly F, Pilarczyk E, Genin A, et al. Heparin-induced thrombocytopenia in severe COVID-19. Circulation. 2020;142(19):1875–7. 10.1161/CIRCULATIONAHA.120.049015 . 32990022 PMC7643786

[22] Sartori M, Cosmi B. Heparin-induced thrombocytopenia and COVID-19. Hematol Rep. 2021. 10.4081/hr.2021.8857 . 33747413 PMC7970398

[23] Kowalska MA, Rauova L, Poncz M. Role of the platelet chemokine platelet factor 4 (PF4) in hemostasis and thrombosis. Thromb Res. 2010;125(4):292–6. 10.1016/j.thromres.2009.11.023 20004006

[24] Rauova L, Poncz M, McKenzie SE, Reilly MP, Arepally G, Weisel JW, et al. Ultralarge complexes of PF4 and heparin are central to the pathogenesis of heparin-induced thrombocytopenia. Blood. 2005;105(1):131–8. 10.1182/blood-2004-04-1544 . 15304392

[25] Greinacher A. Heparin-induced thrombocytopenia. N Engl J Med. 2015;373(3):252–61. 10.1056/NEJMcp1411910 . 26176382

[26] Nguyen TH, Medvedev N, Delcea M, Greinacher A. Anti-platelet factor 4/polyanion antibodies mediate a new mechanism of autoimmunity. Nat Commun. 2017. 10.1038/ncomms14945 . 28530237 PMC5458132

[27] Dawes J, Smith RC, Pepper DS. The release, distribution, and clearance of human β-thromboglobulin and platelet factor 4. Thromb Res. 1978;12(5):851–61. 10.1016/0049-3848(78)90279-7 79232

[28] Khandelwal S, Lee GM, Hester CG, Poncz M, McKenzie SE, Sachais BS, et al. The antigenic complex in heparin-ionduced thrombocytopenia binds to B cells via complement and complement receptor 2 (CD21). Blood. 2016;128(14):1789–99. 10.1182/blood-2016-04-709634 . 27412887 PMC5054694

[29] Staibano P, Arnold DM, Bowdish DME, Nazy I. The unique immunological features of heparin-induced thrombocytopenia. Br J Haematol. 2017;177(2):198–207. 10.1111/bjh.14603 . 28369702

[30] Zheng Y, Wang AW, Yu M, Padmanabhan A, Tourdot BE, Newman DK, et al. B-cell tolerance regulates production of antibodies causing heparin-induced thrombocytopenia. Blood. 2014;123(6):931–4. 10.1182/blood-2013-11-540781 . 24357731 PMC3916881

[31] Madeeva D, Cines DB, Poncz M, Rauova L. Role of monocytes and endothelial cells in heparin-induced thrombocytopenia. Thromb Haemost. 2016;116(5):806–12. 10.1160/TH16-02-0162 . 27487857

[32] Warkentin TE, Basciano PA, Knopman J, Bernstein RA. Spontaneous heparin-induced thrombocytopenia syndrome: 2 new cases and a proposal for defining this disorder. Blood. 2014;123(23):3651–4. 10.1182/blood-2014-01-549741 . 24677540

[33] Greinacher A, Selleng K, Warkentin TE. Autoimmune heparin-induced thrombocytopenia. J Thromb Haemost. 2017;15(11):2099–114. 10.1111/jth.13813 . 28846826

[34] Hippensteel JA, LaRiviere WB, Colbert JF, Langou t-Astri CJ, Schmidt EP. Heparin as a therapy for COVID-19: Current evidence and future possibilities. Am J Physiol Lung Cell Mol Physiol. 2020;319(2):L211–7. 10.1152/ajplung.00199.2020 . 32519894 PMC7381711

[35] Taquet M, Husain M, Geddes J, Luciano S, Harrison Paul. COVID-19 and cerebral venous thrombosis: a retrospective cohort study of 513,284 confirmed COVID-19 cases. OSH Home. 2021. [preprint]. 10.17605/OSF.IO/H2MT7 PMC8324974 34368663

[36] Brodard J, Kremer Hovinga JA, Fontana P, Studt J, Gruel Y, Greinacher A. COVID-19 patients often show high-titer non-platelet-activating anti-PF4/heparin IgG antibodies. J Thromb Haemost. 2021;19(5):1294–8. 10.1111/jth.15262 . 33550713 PMC8013750

[37] Comer SP, Cullivan S, Szklanna PB, Weiss L, Cullen S, Kelliher S, et al. COVID-19 induces a hyperactive phenotype in circulating platelets. PLoS Biol. 2021;19(2):e3001109. 10.1371/journal.pbio.3001109 33596198 PMC7920383

[38] Suzuki K, Okada H, Tomita H, Sumi K, Kakino Y, Yasuda R, et al. Possible involvement of Syndecan-1 in the state of COVID-19 related to endothelial injury. Thromb J. 2021. 10.1186/s12959-021-00258-x . 33504351 PMC7838859

[39] Fraser DD, Patterson EK, Slessarev M, Gill SE, Martin C, Daley M, et al. Endothelial injury and glycocalyx degradation in critically ill coronavirus disease 2019 patients:implications for microvascular platelet aggregation. Crit Care Explor. 2020. 10.1097/CCE.0000000000000194 . 32904031 PMC7449254

[40] Medetalibeyoglu A, Emet S, Kose M, Akpinar TS, Senkal N, Catma Y, et al. Serum Endocan Levels on Admission Are Associated With Worse Clinical Outcomes in COVID-19 Patients: A Pilot Study. Angiology. 2021;72(2):187–93. 10.1177/0003319720961267 . 32969233

[41] Woodruff MC, Ramonell RP, Nguyen DC, Cashman KS, Saini AS, Haddad NS, et al. Extrafollicular B cell responses correlate with neutralizing antibodies and morbidity in COVID-19. Nat Immunol. 2020;21(12):1506–1516. 10.1038/s41590-020-00814-z . 33028979 PMC7739702

[42] Boilard E, Paré G, Rousseau M, Cloutier N, Dubuc I, Lévesque T, et al. Influenza virus H1N1 activates platelets through FcγRIIA signaling and thrombin generation. Blood. 2014;123(18):2854–63. 10.1182/blood-2013-07-515536 . 24665136

[43] Hottz ED, Oliveira MF, Nunes PCG, Nogueira RMR, Valls-de-Souza R, Da Poian AT, et al. Dengue induces platelet activation, mitochondrial dysfunction and cell death through mechanisms that involve DC-SIGN and caspases. J Thromb Haemost. 2013;11(5):951–962. 10.1111/jth.12178 . 23433144 PMC3971842

[44] Greinacher A, Thiele T, Warkentin TE, Weisser K, Kyrle PA, Eichinger S. Thrombotic thrombocytopenia after ChAdOx1 nCov-19 vaccination. N Engl J Med. 2021. 10.1056/NEJMoa2104840 33835769 PMC8095372

[45] Schultz NH, Sørvoll IH, Michelsen AE, Munthe LA, Lund-Johansen F, Ahlen MT, et al. Thrombosis and thrombocytopenia after ChAdOx1 nCoV-19 Vaccination. N Engl J Med. 2021. 10.1056/NEJMoa2104882 33835768 PMC8112568

[46] Scully M, Singh D, Lown R, Poles A, Solomon T, Levi M, et al. Pathologic Antibodies to platelet factor 4 after ChAdOx1 nCoV-19 Vaccination. N Engl J Med. 2021. 10.1056/NEJMoa2105385 33861525 PMC8112532

[47] See I, Su JR, Lale A, Woo EJ, Guh AY, Shimabukuro TT, et al. US case reports of cerebral venous sinus thrombosis with thrombocytopenia After Ad26.COV2.S vaccination, March 2 to April 21, 2021. JAMA. 2021. 10.1001/jama.2021.7517 . 33929487 PMC8087975

[48] Greinacher A, Mayerle J, Aebischer A, Warkentin TE, Muenchhoff M, Hellmuth JC, et al. Anti-SARS-CoV-2 spike protein and anti-platelet factor 4 antibody responses induced by COVID-19 disease and ChAdOx1 nCov-19 vaccination. ResearchSquare. 2021. [preprint]. 10.21203/rs.3.rs-404769/v1

[49] Greinacher A, Handtke S, Lalk M, Methling K, Beer M, Institut F-L, et al. Towards understanding ChAdOx1 nCov-19 vaccine-induced immune thrombotic thrombocytopenia (VITT). ResearchSquare. 2021. [preprint]. 10.21203/rs.3.rs-440461/v1

[50] Othman M, Labelle A, Mazzetti I, Elbatarny HS, Lillicrap D. Adenovirus-induced thrombocytopenia: The role of von Willebrand factor and P-selectin in mediating accelerated platelet clearance. Blood. 2007;109(7):2832–2839. 10.1182/blood-2006-06-032524 . 17148587

[51] Mercier S, Gahéry-Segard H, Monteil M, Lengagne R, Guillet J-G, Eloit M, et al. Distinct roles of adenovirus vector-transduced dendritic cells, myoblasts, and endothelial cells in mediating an immune response against a transgene product. J Virol. 2002;76(6):2899–2911. 10.1128/jvi.76.6.2899-2911.2002 . 11861857 PMC136003

[52] Lei Y, Zhang J, Schiavon CR, He M, Chen L, Shen H, et al. SARS-CoV-2 spike protein impairs endothelial function via downregulation of ACE2. Circ Res. 2021. 10.1161/circresaha.121.318902 . 33784827 PMC8091897

[53] Nuovo GJ, Magro C, Shaffer T, Awad H, Suster D, Mikhail S, et al. Endothelial cell damage is the central part of COVID-19 and a mouse model induced by injection of the S1 subunit of the spike protein. Ann Diagn Pathol. 2021. 10.1016/j.anndiagpath.2020.151682 . 33360731 PMC7758180

[54] Liu L, Chopra P, Li X, Bouwman K, Tompkins SM, Wolfert M, et al. Heparan sulfate proteoglycans as attachment factor for SARS-CoV-2. bioRxiv. 2020. [preprint]. 10.1101/2020.05.10.087288 34235261 PMC8227597

